# Efficacy and safety profile of statins in patients with cancer: a systematic review of randomised controlled trials

**DOI:** 10.1007/s00228-020-02967-0

**Published:** 2020-07-28

**Authors:** John P. Thomas, Yoon K. Loke, Leo Alexandre

**Affiliations:** 1 Norwich Medical School, University of East Anglia, Norwich, NR4 7TJ UK; 2 Department of Gastroenterology, Norfolk and Norwich University Hospital, Norwich, NR47UY UK

**Keywords:** Statin, HMG-CoA, Clinical trials, Cancer, Adverse effects

## Abstract

**Purpose:**

A growing body of preclinical and observational research suggests that statins have potential as a therapeutic strategy in patients with cancer. This systematic review of randomised controlled trials (RCTs) in patients with solid tumours aimed to determine the efficacy of statin therapy on mortality outcomes, their safety profile and the risk of bias of included studies.

**Methods:**

Full-text articles comparing statin therapy versus control in solid tumours and reporting mortality outcomes were identified from Medline and Embase from conception to February 2020. A systematic review with qualitative (primarily) and quantitative synthesis was conducted. This systematic review was prospectively registered (Prospero registration CRD42018116364).

**Results:**

Eleven trials of 2165 patients were included. Primary tumour sites investigated included lung, colorectal, gastro-oesophageal, pancreatic and liver. Most trials recruited patients with advanced malignancy and used sub-maximal statin doses for relatively short durations. Aside from one trial which demonstrated benefit with allocation to pravastatin 40 mg in hepatocellular carcinoma, the remaining ten trials did not demonstrate efficacy with statins. The pooled hazard ratio for all-cause mortality with allocation to pravastatin in patients with hepatocellular carcinoma in two trials was 0.69 (95% confidence interval CI 0.30–1.61). Study estimates were imprecise. There were no clinically important differences in statin-related adverse events between groups. Overall, included trials were deemed low risk of bias.

**Conclusion:**

The trial evidence is not sufficiently robust to confirm or refute the efficacy and safety of statins in patients with solid malignant tumours. Study and patient characteristics may explain this uncertainty. The potential role of high-dose statins in adjuvant settings deserves further research.

**Electronic supplementary material:**

The online version of this article (10.1007/s00228-020-02967-0) contains supplementary material, which is available to authorized users.

## Background

Hydroxy-3-methylglutaryl-CoA (HMG-CoA) inhibitors, better known as statins, are a class of lipid-lowering agents that are highly effective and used widely in clinical practice for the primary and secondary prevention of cardiovascular disease [1]. Statins inhibit the rate-limiting step of the mevalonate pathway, a ubiquitous metabolic cascade which plays an essential role in the synthesis of downstream sterol (e.g. cholesterol) and non-sterol isoprenoids [2]. There is growing evidence that a number of these biologically active intermediates exert functions which have direct relevance to cancer biology, with roles in proliferative signalling, cell-cycle regulation, angiogenesis, and metastases [3]. Interest in the potential of statins to prevent and treat cancer has grown over the last three decades.

In vitro studies have demonstrated that statins inhibit proliferation, induce apoptosis and limit invasiveness in numerous malignancies, and have demonstrated the functional relevance of mevalonate pathway intermediates in these observations [4–6]. Mutant TP53, the most frequently mutated gene in cancer [7, 8] and consistently associated with poor prognosis [9], has been shown to upregulate transcription of mevalonate pathway products to sustain malignant proliferation [10], a pathway potently inhibited by statins. Furthermore, statins have been shown to selectively destabilise mutant TP53 protein [11]. Preclinical in vivo studies have demonstrated statins effectively inhibit growth of established tumours with no noticeable effect on normal tissues [11, 12]. These preclinical observations underscore the potential for statins as a viable therapeutic strategy in human malignancy.

The most recent systematic review of observational research included 95 cohorts with over 1.1 million cancer patients and demonstrated post-diagnostic statin use was associated with a significant reduction in all-cause mortality (HR 0.70, 95% CI 0.66–0.74 pooled from 55 studies), with broadly similar effect sizes for progression-free survival, cancer-specific mortality and disease-free survival [13]. However, to varying degrees, studies were potentially susceptible to selection bias, immortal-time bias and confounding. Nevertheless, compared with studies with a higher risk of bias (≤ 8 points on a 6-item scale [14]), effect sizes of those with a lower risk of bias (> 8 points) were attenuated, however remained statistically significant. While preclinical and epidemiological evidence is encouraging, causality remains to be established. To determine whether statins are an effective therapeutic option for specific cancers, evidence from well-designed, sufficiently powered, randomised controlled trials (RCTs) are required.

A series of trials have assessed the efficacy and safety of statins in patients with solid tumours; however, there remains considerable uncertainty, and the justification for further trials has been questioned [15]. The conduct of future trials should be reliably informed by critical appraisal of existing randomised studies in patients with cancer. Therefore, we undertook a systematic review of statins in patients with any malignancy to assess the current state of evidence from RCTs. Specifically, in patients with solid tumours, we aimed to determine (i) the efficacy of statin therapy on mortality outcomes, (ii) the safety profile of statins, and (iii) the risk of bias in RCTs of statin therapy.

## Methods

This systematic review was registered (CRD42018116364) on the PROSPERO database and conducted in accordance with the PRISMA (Preferred Reporting Items for Systematic Reviews and Meta-Analyses) guidelines [16].

### Search strategy

We sought relevant published articles by searching MEDLINE (1948 onwards) and Embase (1980 onwards) (Supplementary Table 1) using the OVID interface and manual searches of reference lists of any systematic reviews identified by the previous step. We used the following search terms to search each database: hydroxymethylglutaryl-CoA reductase inhibitors, statin, cancer, carcinoma, neoplasms, malignancy and randomised controlled trial. The literature search was limited to the English language and human subjects. Searches were completed in Feb 2020.

### Eligibility criteria

Only RCTs satisfying the following eligibility criteria were included in the systematic review: (i) statin therapy was the intervention, either given alone or in combination with a co-intervention across trial arms; (ii) at least one trial group received placebo, no statin or standard care alone; (iii) participants were diagnosed with a malignant solid tumour prior to enrolment; and (iv) overall survival (OS), progression-free survival (PFS) or response rate (RR) were reported outcomes. No restrictions were placed on the statin administered, posology, frequency or duration of administration. No restrictions were placed on length of follow-up. Two reviewers (JPT and LA) independently screened abstracts and selected full-text articles for inclusion based on the above criteria. Discrepancies were resolved through discussion among two or more reviewers.

### Data extraction and quality assessment

Two reviewers (JPT and LA) independently extracted data from each selected article for study characteristics (location, setting, number of randomised patients, recruitment period, primary cancer site, intervention, duration of statin therapy, concomitant therapy and reported outcome measures); patient characteristics at enrolment (number of patients allocated to active and control groups, age, gender, cancer stage and Eastern Cooperative Oncology Group [ECOG] performance status); study outcomes (reported median overall and progression-free survival in allocated groups with corresponding hazard ratios (and confidence intervals) and reported response rates (%) in each group); and toxicity profile. For continuous participant characteristics and outcomes, we extracted means (with corresponding standard deviations) and medians (with corresponding ranges) as appropriate in each arm. To assist the comparison of statin type and posology used between studies, the defined daily dose (DDD) for each trial was calculated [17]. The DDD is a standardised measure of drug exposure relative to the assumed average maintenance dose per day for a drug used, for its main indication in adults was as defined by the World Health Organization. For example, a single dose of simvastatin 30 mg or atorvastatin 20 mg is equivalent to 1 DDD. Two reviewers (JPT and LA) used the Cochrane risk of bias tool to assess internal validity of each eligible study across seven items: random sequence generation, allocation concealment, blinding of participants and personnel, blinding of outcome assessment, incomplete outcome data, selective reporting and other sources of bias [18]. Given the outcomes of interest were objective (e.g. all-cause mortality), open-label study designs, where applicable, were deemed to pose minimal risk of bias for the domains of “blinding of participants and personnel”, and “blinding of outcome assessment”. Discrepancies were resolved through consensus discussion between reviewers. We contacted authors for additional information where required.

### Study outcomes

The primary outcome was overall survival (OS), defined as the time from randomisation to death from any cause [19]. Secondary outcomes were (i) progression-free survival (PFS), defined as time from randomisation to first observed cancer progression or death; (ii) response rate (RR), defined as the proportion of patients with tumour size reduction of a predefined amount and for a minimum time period [19]; and (iii) toxicity (proportions of grade 3–5 and separately statin-related adverse events in each group).

### Statistical analysis

From the outset, we decided it would be inappropriate to conduct a quantitative meta-analysis comprising trials with different primary cancers as any resultant summary effect size estimate for mortality outcomes would be difficult to interpret. This is because each distinct cancer has disparate biology, behaviour, prognosis, treatments and responsiveness to therapy. Furthermore, while the mevalonate pathway is ubiquitous to all eukaryotes and will be functional in malignancy, there is insufficient evidence at present to suggest a universally consistent role in effecting cancer prognosis. As a result, we primarily undertook a qualitative assessment of included trials to critically review the study characteristics, participant characteristics, mortality and safety outcomes of eligible studies. We performed a quantitative meta-analysis, where possible, of any trials in patients with the same primary cancer.

Summary study characteristics were calculated and weighted by sample size for gender, cancer stage and ECOG performance status. Where *p* values were not provided in original study reports for comparisons between intervention and control arm for overall response rate, we calculated these with extracted categorical data using the chi-squared test or Fisher’s exact test, as appropriate. Meta-analysis of trials involving patients with the same primary cancer was performed to quantify the association between statin use and overall survival. Effect estimates were pooled by the inverse of their variance and are presented as pooled hazard ratios (HRs) with corresponding 95% CIs. Due to differences in recruited study populations, concomitant therapies and intervention protocols, we utilised a random-effects meta-analysis using the method of DerSimonian and Laird [20]. Heterogeneity was estimated using the Cochrane’s *Q* and *I*^2^ statistics. A two-tailed *p* value of less than 0.05 was defined as statistically significant for all analyses apart from Cochrane’s *Q* test for heterogeneity where a *p* value of 0.10 was selected as the threshold of significance. Results of this meta-analysis were illustrated by means of a forest plot. Analyses were performed with STATA version 15.1 (StataCorp LP, College Station, TX, USA).

## Results

### Search and selection of studies

**Fig. 1:**
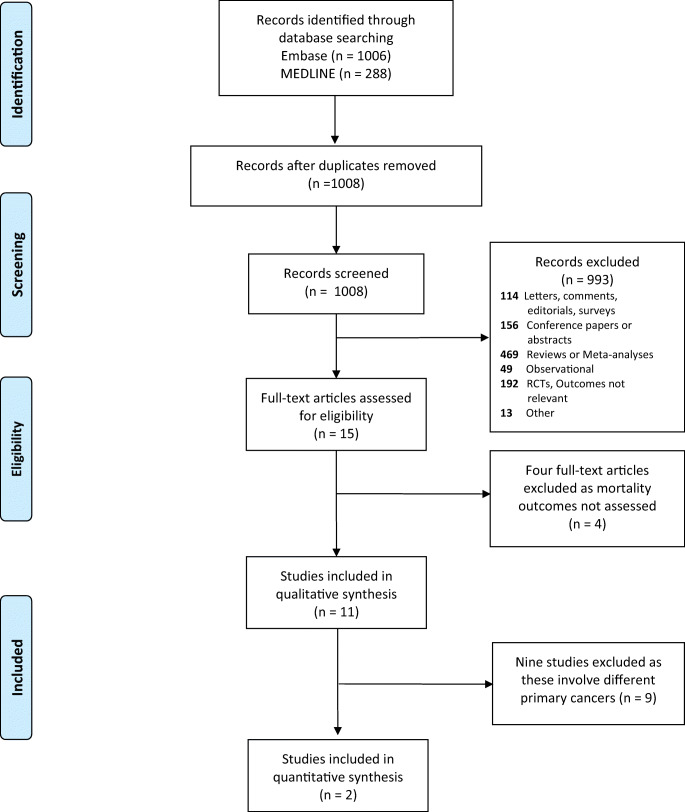
PRISMA flow diagram

Among 1008 articles identified from the literature search, 15 full-text articles were assessed for eligibility, of which eleven were ultimately eligible for inclusion (Fig. 1) [21–31]. The four excluded articles met all inclusion criteria except for the outcomes of interest, instead focusing on surrogate outcomes [32–35].

### Study characteristics

The characteristics of selected studies are shown in Table 1. Four were phase III studies [22, 24, 26, 27] and the remainder were phase II/pilot/feasibility trials. Of the eleven RCTs, six originated from East Asia [23, 26–29, 31]. Four studies were performed in Europe [21, 22, 24, 30] and one in Egypt [25]. All studies were conducted in hospital-based settings. In total, 2165 participants were recruited across all trials. Four studies were conducted at a single site and included between 30 and 106 participants [25, 29–31]. The largest trial included 846 patients (LUNGSTAR) across 91 UK centres [24]. The gastro-intestinal tract and accessory digestive organs were the primary site examined in seven trials, including cancers of the gastro-oesophageal junction/stomach [27, 30], oesophagus/gastro-oesophageal junction [21], pancreas [26], liver [22, 31] and colorectum [26]. The remaining studies separately recruited patients with small cell lung cancer [24], non-small cell lung cancer [23, 29] and brain metastases (with various primary tumour sites) [25]. Eight of the trials explicitly excluded prior/current statin users [21, 22, 24–26, 28–30], and no trials reported the proportion of prior/current users among the randomised population. The intervention arm in seven studies was simvastatin [21, 23, 25–29] and in four studies was pravastatin [22, 24, 30, 31]. The highest DDD used in one study was 2.67 [25] and in the remaining ten studies was 1.33. Open-label statins were administered in six studies [22, 23, 25, 29–31], and identical matched placebo was used in those remaining. Reported median duration of statin therapy administration was 3–8.6 months [22, 24, 26, 27, 29]. One trial of pravastatin in hepatocellular carcinoma administered statins for a mean of 16.5 months [31]. Concomitant chemo/radiotherapy was administered in all but one trial [21].

**Table 1:** Characteristics of selected randomised controlled trials

Trial	Location	Centre(s)	Number of patients	Recruitment period	Cancer	Intervention (DDD)	Duration of statin therapy (months)	Previous cancer therapy	Concomitant therapy	Outcomes
Alexandre et al. [21]	UK	3	32	Oct 2014–July 2016	Oesophageal/GOJ adenocarcinoma	Simvastatin 40 mg (1.33) or Placebo	9.6	Yes^b^	Nil	Retention, Absorption, Adherence, OS, PFS, QoL
Jouve et al. [22]	France	61	323	March 2010–Nov 2013	Hepatocellular Carcinoma	Pravastatin 40 mg (1.33), open label	4.1^Md^	Yes	Chemotherapy (S)	OS, PFS, TTP, TTF, QoL
Lee et al. [23]	South Korea	2	68	Nov 2012–Sept 2015	NA Non-Small Cell Lung Cancer	Simvastatin 40 mg (1.33), open label	NS	Yes^c^	Afatinib	OS, PFS, RR
Seckl et al. [24]	UK	91	846	Feb 2007–Jan 2012	Small Cell Lung Cancer	Pravasatatin 40 mg (1.33) or placebo	8.6^Md^	Nil	Chemotherapy (ET + C or CB)	OS, PFS, RR
El-Hamamsy et al. [25]	Egypt	1	50	April 2014–Oct 2015	Brain metastases (various primaries^a^)	Simvastatin 80 mg (2.67), open label	0.5	NS	Whole brain radiotherapy	OS, PFS, Rad R
Lim et al. [26]	South Korea	5	269	April 2010–July 2013	Colorectal Cancer	Simvastatin 40 mg (1.33) or Placebo	3–4.5^Md^	Yes^c^	Chemotherapy (XELIRI/FOLFIRI)	OS, PFS, RR, TTP
Kim et al. [27]	South Korea	9	244	Feb 2009–Nov 2014	Gastric/GOJ adenocarcinoma	Simvastatin 40 mg (1.33) or placebo	4.43^Md^	Yes^d^	Chemotherapy (C + X)	OS, PFS, RR
Hong et al. [28]	South Korea	4	114	Dec 2008–April 2012	Pancreatic cancer	Simvastatin 40 mg (1.33) or Placebo	NS	Nil	Chemotherapy (GC)	OS, RR, TTP, DCR
Han et al. [29]	South Korea	1	106	May 2006–Sept 2008	Non-Small Cell Lung Cancer	Simvastatin 40 mg (1.33), open label	8.6^Md^	Yes	Chemotherapy (GF)	OS, PFS, RR
Konings et al. [30]	Netherlands	1	30	Feb 2005–May 2009	Gastric adenocarcinoma	Pravastatin 40 mg (1.33), open label	3.5^Mx^	Nil	Chemotherapy (E, C + CB)	OS, PFS, RR
Kawata et al. [31]	Japan	1	83	Feb 1990–Jan 1993	Hepatocellular Carcinoma	Pravastatin 40 mg (1.33), open label	16.5^Me^	Nil	TACE +5FU	OS

*5FU* 5-Flurouracil, *C* cisplatin, *CB* carboplatin, *DCR* disease control rate, *DDD* defined daily dose, *DX* dexamethasone, *E* epirubicin, *ET* etoposide, *FOLFIRI* 5-fluorouracil and irinotecan, *GC* gemcitabine, *GOJ* gastro-oesophageal junction, *GF* gefitanib, *OS* overall survival, *Md* median, *Me* mean, *Mx* maximum, *NA* non-adenocarcinomatous, *NS* not stated, *PFS* progression-free survival, *QoL* quality of life, *RR* response rate, *Rad R* radiological response, *S* sorafenib, *TACE* transcatheter arterial chemoembolisation, *TTF* time to treatment failure, *TTP* time to progression, *THL* thalidomide, *X* capecitabine, *XELIRI regimen* capecitabine plus irinotecan

^a^Primary cancers were mostly breast and lung cancers (in 88% of patients)

^b^94% patients received prior chemotherapy

^c^All patients received prior chemotherapy

^d^36.6% in statin group and 45.1% in control group received prior chemotherapy

### Patient characteristics

The mean age of recruited participants between trials was between 53 and 68 years (Supplementary Table 2) and was generally well balanced between groups. Of all recruited participants, 64.5% were male. Gender was generally well balanced between groups; however, there were numerical differences (≥ 10%) for three small trials [23, 25, 30]. All but one trial [21] included patients with metastatic disease at enrolment. Eight trials reported exact proportions with metastatic disease [21, 22, 24, 26–29, 31], comprising 2035 recruited participants, of which 65% had metastases (Table 2). Disease staging appeared well balanced between groups. Of nine trials which reported ECOG performance status [21, 22, 24, 26–30], including 2032 patients, 87% were status 0–1, and 13% were status 2–3. ECOG performance status appeared well balanced between groups.

**Table 2:** Cancer stage and performance status

Trial	Cancer type	Stage of Cancer		ECOG 0 or 1 in statin group	ECOG 0 or 1 in control group	ECOG 2 or 3 in statin group	ECOG 2 or 3 in control group
		Statin group	Control group				
Alexandre et al. [21]	Oesophageal/GOJ adenocarcinoma	Up to stage 3		93.8%	100%	6.2%	0%
Jouve et al. [22]	Hepatocellular Carcinoma	Child-Pugh A Extra-hepatic Metastatic disease: 29.0%	Child-Pugh A Extra-hepatic metastatic disease: 30.4%	95.7%	95%	4.3%	5%
Lee et al. [23]	Non-adenocarcinomatous Non-small cell lung cancer	Stage 3B/4^b^		75%	75%	25%	25%
Seckl et al. [24]	Small cell lung cancer	Limited disease^c^: 43.4%	Limited disease^c^: 42.7%,	75.6%	75.2%	24.4%	24.8%
		Extensive disease^c^: 56.6%	Extensive disease^c^: 49.5%				
El-Hamamsy et al. [25]	Brain metastases (various primaries^a^)	Stage 4		NS^e^	NS	NS	NS
Lim et al. [26]	Colorectal Cancer	Stage 4^b^		98.5%	98.5%	1.5%^g^	1.5%^g^
Kim et al. [27]	Gastric/GOJ adenocarcinoma	Stage 4^b^		99.2%	96.7%	0.8%	3.3%
Hong et al. [28]	Pancreatic cancer	Locally advanced disease: 12%	Locally advanced disease: 12.5%	100%	100%	0%	0%
		Metastatic disease: 88%	Metastatic disease: 87.5%				
Han et al. [29]	Non-small cell lung cancer	Stage 3b^b^: 6%, stage 4: 94%	Stage 3b^b^: 11%, stage 4: 89%	94%	89%	6%	11%
Konings et al. [30]	Gastric adenocarcinoma	≥ 43% metastatic disease		86.7%	100%	13.3%^g^	0%
Kawata et al. [31]	Hepatocellular carcinoma	Stage 2–3^d^: 73%, stage 4: 27%	Stage 2–3^d^: 69%, stage 4: 31%	NS^f^	NS	NS	NS

*ECOG* Eastern Cooperative Oncology Group, *GOJ* gastro-oesophageal, *NS* not stated

^a^Primary cancers were mostly breast and lung cancers (in 88% of patients)

^b^American Joint Committee on Cancer TNM staging

^c^Veterans Administration Lung Study Group Staging

^d^Primary Liver Cancer Study Group of Japan staging

^e^36% in the statin group and 28% in the control group had a Karnofsky performance scale score of > = 70%

^f^83% in the statin group and 86% in the control group had a Karnofsky performance scale score of > 70%

^g^No ECOG > 2 patients

^f^83% in the statin group and 86% in the control group had a Karnofsky performance scale score of > 70%

### Mortality outcomes

Two trials investigated the effect of pravastatin 40 mg in patients with advanced hepatocellular carcinoma [22, 31]. Allocation to pravastatin therapy was associated with significantly improved overall survival in one of these studies only [31]: median survival was 18 months in the pravastatin group and 9 months in the control group (HR 0.42, 95% CI 0.20–0.83). Meta-analysis of overall survival with pravastatin in both these trials revealed a HR of 0.69 (95% CI: 0.30–1.61) which was not statistically significant (*p* = 0.392) (Supplementary Fig. 1). The Cochrane *Q* test (*p* = 0.024) and *I*^2^ statistic (80.5%) demonstrated a statistically significant degree of heterogeneity (*p* < 0.10). None of the other included trials demonstrated significant improvements in overall survival with statins, including for small-cell lung cancer, non-small cell lung cancer, oesophageal/GOJ/gastric cancers, colorectal cancer and pancreatic cancer. No improvements in progression-free survival were observed with allocation to statins individually in nine studies (*n* = 2050) in which this outcome was reported [22–30]. There were no significant differences in overall response rate for the eight studies (*n* = 1727) reporting this outcome [23–30].

### Safety profile

Five trials reported grades 3–5 adverse events. None of these trials demonstrated significant differences in grades 3–5 adverse events between statin and control group (*n* = 1497) outcome [21, 24, 26, 27, 29] (Supplementary Table 3). Statin-related adverse events (myalgia/myopathy or abnormal alanine aminotransferase/aspartate aminotransferase or elevated creatine phosphokinase) were similar in proportion between groups in all nine studies reporting these outcomes [21–28, 30]. Most trials had small sample sizes and may have been inadequately powered to detect clinically relevant differences in adverse events if they existed (Table 3).

**Table 3:** Major study outcomes

	Median OS (months)			Median PFS (months)			Overall response rate (%)		
Trial	Statin group	Control group	HR (95% CI), *p* value	Statin group	Control group	HR (95% CI), *p* value	Statin	Control	*p* value
Alexandre et al. [21]	NS	NS	HR 1.56 (0.14-17.28), p = 0.716	NS	NS	HR 0.78 (0.11-5.61), p = 0.807	NS	NS	NS
Jouve et al. [22]	10.7 (7.7–14.3)	10.5 (8.2–12.4)	1.00 (0.79–1.28), *p* = 0.975	5.0 (3.4–6.0)	4.4 (3.3–5.6)	1.00 (0.80–1.25), *p* = 0.986	NS	NS	NS
Lee et al. [23]	10.0 (6.4–13.8)	7.0 (6.1–7.9)	1.03 (0.58–1.80), *p* = 0.466	1.0 (0.5–1.4)	3.6 (3.0–4.1)	1.38 (0.84–2.29), *p* = 0.898	5.70%	9.40%	0.43
Seckl et al. [24]	10.7	10.6	1.01 (0.88–1.16), *p* = 0.90	7.7	7.3	0.98 (0.85–1.13), *p* = 0.81	69%	69.10%	0.963^c^
El-Hamamsy et al. [25]	3.4 (0.69–6.01)	3 (2.46–3.54)	NS, *p* = 0.880	1.6 (0.68–2.52)	1.47 (0.91–2.02)	NS, *p* = 0.392	78.6%^b^	60%^b^	0.427
Lim et al. [26]	15.3 (12.1–18.5)	19.2 (16.8–21.6)	NS, p = 0.826^a^	5.9 (4.5–7.3)	7 (5.4–8.6)	1.03 (0.77–1.37), *p* = 0.858	11.90%	11.80%	1
Kim et al. [27]	11.6 (9.2–13.9)	11.5 (9.9–13.1)	NS, *p* = 0.818	5.2 (4.3–6.1)	4.6 (3.5–5.7)	0.93 (0.68–1.26), *p* = 0.664	27.50%	29.00%	0.936
Hong et al. [28]	6.6 (4.4–8.2)	8.7 (4.8–12.6)	NS, p = 0.98	2.4 (0.7–4.1)	3.6 (3.1–4.1)	NS, 0.903	6.90%	14.30%	0.23
Han et al. [29]	13.6 (7.1–20.1)	12 (7.8–16.2)	0.88 (0.57–1.35), *p* = 0.491	3.3 (1.4–5.2)	1.9 (1.0–2.8)	0.891 (0.60–1.32), *p* = 0.549	38.50%	31.50%	0.666
Konings et al. [30]	8 (3.02–12.98)	6 (4.93–7.08)	NS	6 (3.39–8.61)	5 (3.83–6.17)	NS	33.30%	46.70%	0.473
Kawata et al. [31]	18	9	0.42 (0.20–0.83), *p* = 0.006	NS	NS	NS	NS	NS	NS

*NS* not stated, *OS* overall survival, *PFS* progression-free survival

Figures in parenthesis indicate 95% confidence intervals

^a^*p* value calculated using log-rank test

^b^Radiological response

^c^*p* value calculated using chi-squared test

Figures in parenthesis indicate 95% confidence intervals

### Risk of bias

**Fig. 2:**
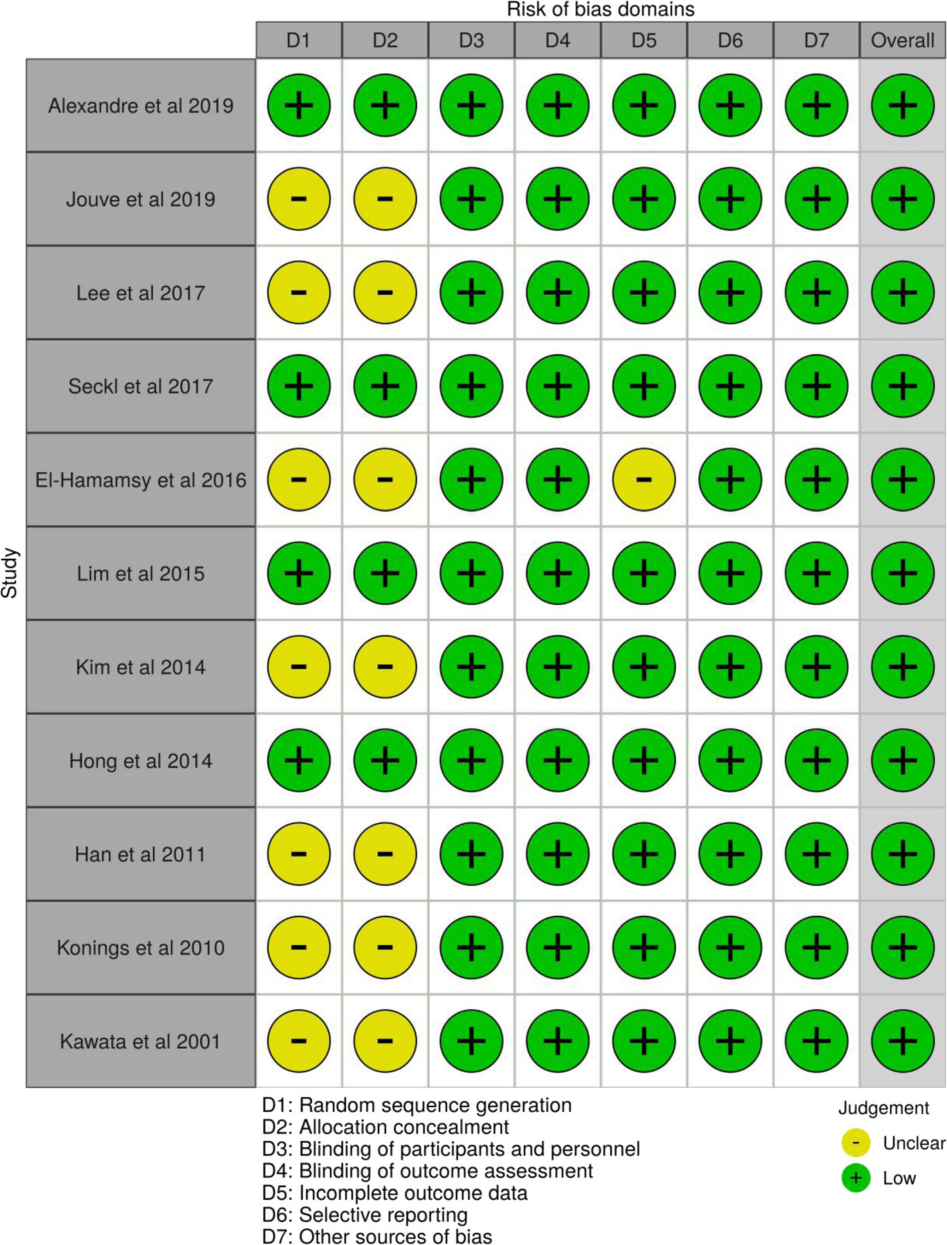
Risk of bias of selected studies using the Cochrane risk of bias tool

Figure 2 shows the assessment of risk of bias in the included trials as per the Cochrane risk of bias tool, illustrated using the robvis application [36]. Four trials reported random sequence generation and allocation concealment adequately [21, 24, 26, 28], while this was insufficiently reported in the remaining seven. While six trials were open-label studies, any deviations from intended intervention were unlikely to impact on the outcome and therefore were deemed at low risk of performance bias [22, 23, 25, 29–31]. Risk of detection bias for all trials overall was determined to be low given that knowledge of statin allocation (where applicable to open-label studies) would seem unlikely to bias reported outcomes not involving subjective judgement, such as mortality outcomes or measures of treatment response. All trials were deemed to be at low risk of selective reporting.

## Discussion

In summary, this systematic review included eleven trials of statin therapy in 2165 patients with solid tumours in total, including small cell lung cancer (*n* = 846), non-small cell lung cancer (*n* = 106 and *n* = 68), colorectal cancer (*n* = 269), gastric adenocarcinoma (*n* = 244 and *n* = 30), oesophageal adenocarcinoma (*n* = 32), pancreatic cancer (*n* = 114), hepatocellular cancer (*n* = 83 and *n* = 323) and patients with brain metastases (from mainly breast and lung primaries) (*n* = 50). Most patients recruited had advanced malignancy and received concomitant palliative chemotherapy. Most patients received 40 mg of simvastatin or pravastatin (1.33 DDD), and typically for short durations (on average fewer than 9 months). Most trials did not demonstrate significant improvements in overall survival (aside from one trial of pravastatin 40 mg in hepatocellular carcinoma [31]), and no trials reported improvements in progression-free survival or overall response rate. Meta-analysis of the two trials involving pravastatin 40 mg in advanced hepatocellular cancer [22, 31] revealed no significant improvements in overall survival (Supplementary Fig. 1). There was no indication in any trial of an increased rate of adverse events in those allocated to statins. Overall, included trials were deemed to be at low risk of bias using the Cochrane risk of bias tool [18].

### Comparison with previous work

This is the second systematic review of RCTs to examine both the clinical efficacy and safety profile of statins in patients with solid tumours. The first included a meta-analysis of eight RCTs included in this systematic review [37]. This review provided a brief description of study characteristics and the overwhelming focus was on quantitative synthesis of the effect of statins on OS, PFS, RR and adverse events. In contrast, our review is primarily a qualitative synthesis of included trials and provides more detail regarding important characteristics relating to included studies (country, blinding, duration of statin therapy, DDD) and participants (demography, cancer staging, performance status) to aid interpretation. Another more recent systematic review focused on a meta-analysis of nine of the included RCTs in our review to examine the effect of allocation to statins on OS and PFS [38]. As previously stated, we deliberately did not conduct a meta-analysis of all RCTs given irreconcilable heterogeneity of included studies and uncertainty surrounding the assumption of a uniform treatment effect, with resultant difficulties in interpretation of summary estimates.

The cholesterol treatment trialists’ collaboration individual patient data (IPD) meta-analysis of 22 RCTs of statin vs. control (primary or secondary prevention of cardiovascular disease, *n* = 134,537) and 5 RCTs of high-dose vs. low-dose statins (secondary prevention, *n* = 39,612) demonstrated no evidence of reduced incident cancer overall (RR 1.00, 95% CI 0.96–1.04) or related cancer-specific mortality (RR 0.98, 95% CI 0.92–1.05) for those allocated to the active arm [39]. No significant associations for mortality were demonstrated individually for any of the 23 primary sites examined. However, only cancers diagnosed after randomisation were considered (1.4% developed cancer per year after randomisation), and it is not clear how many of these patients were receiving study drug from the point of cancer diagnosis. It is therefore difficult to make inferences of the effect of allocation to statins on mortality outcomes in patients with cancer from this IPD meta-analysis.

## Limitations

It is possible that statins do not exert clinically relevant effects in patients with solid tumours; however, other explanations for the divergence of trial evidence from the promising pre-clinical and epidemiological data deserve consideration. Of included studies, only four were phase III studies, and the remaining seven were not powered to detect significant differences in mortality outcomes. Of the phase III studies, three [22, 26, 27] were powered to detect relatively large effect sizes (HR 0.74, HR 0.65, and HR 0.67 respectively) and were at risk of type II error should the actual effect sizes have been more conservative. The largest trial to date in small cell lung cancer (*n* = 846) was powered to detect a HR of 0.82 [24]. Treatment response to statins could feasibly differ between palliative and adjuvant settings, depending on their primary mechanism of action in individual tumour types (for example, a primary effect on inhibition of metastases as seen in colorectal cancer may favour response in the adjuvant setting [40]) and the influence of baseline tumour burden. All but one trial included patients with metastatic disease at baseline (65% of participants overall where reported); in such patients with poor prognosis in receipt of statins for short durations, precluding a marked cytotoxic effect of statins (which would seem unlikely), it may not be possible to elicit or demonstrate treatment response. Furthermore, it is difficult to generalize these trial findings to the adjuvant setting. Although an effective statin dose has yet to be defined in the setting of cancer therapy, and may differ from the licenced doses prescribed for the prevention of cardiovascular disease, the dose of statins assessed in these trials may have been insufficient. All trials used statins at sub-maximal doses (ten with a DDD of 1.33 and one with a DDD of 2.67); higher doses (e.g. atorvastatin 80 mg—DDD 4) are clinically licenced in cardiovascular prevention [41] and could be investigated in a trial. Stratification of effect sizes according to statin type, dose (as defined by DDD) and intended duration of therapy may have been informative; however, such comparisons would have included trials with different primary sites in each strata, and the resulting estimates and tests for interaction would have been difficult to interpret. It is unclear whether statin use prior to randomisation is an effect modifier for the association between statin allocation and mortality outcomes, as most studies excluded prior/current statin use; and those studies which did not specifically exclude such users did not report the proportion of existing users in the randomised population.

### Recommendations

Given the imprecise estimates for efficacy and the limitations of previous trials discussed above, the current trial evidence base does not preclude the conduct of future statin trials in patients with solid malignancies. Further definitive phase III trials are required to determine the efficacy and safety profile of statins in individual tumour types, provided there exists sufficient scientific justification for their conduct: including the proposed mechanism of action applicable to underlying tumour biology and the relevance of the pharmacokinetic properties of the selected statin. High-dose statin therapy should be considered to maximize the probability of observing clinically relevant effects: given the dose-dependent effects of statins in pre-clinical research [42] and trial data for their current licenced indications [41]. Future trials should be adequately powered to detect more conservative effect sizes than previously examined; indeed, relatively small clinically significant differences in primary outcomes may be justifiable given that statins are easily administered, low-cost medications with a favourable safety profile when used for their licenced indications [43]. Investigators should consider the merits of investigating statins in the adjuvant setting, where there is mounting pre-trial evidence [44]. Future trials should ideally collect blood and fresh frozen tissue to permit translational research studies including biomarkers predictive of treatment response.

## Conclusions

Overall, the trial evidence is not sufficiently robust to confirm or refute the efficacy and safety of statins in addition to the current standard of care in patients with solid malignant tumours. Most trials were not adequately powered to detect more conservative differences in efficacy outcomes, and statins were administered for short durations at submaximal doses in patients with predominantly advanced malignancy. Based on this evidence, it may be premature to disregard a potential beneficial role of statins in cancer therapy and there is insufficient evidence to preclude the conduct of future trials. The potential role of high-dose statins in adjuvant settings deserves further research.

## Electronic supplementary material

Supplementary Table 1 (DOCX 13 kb)

Supplementary Table 2 (DOCX 16 kb)

Supplementary Table 3 (XLSX 11 kb)

Supplementary Fig. 1 (DOCX 970 kb)

## Data Availability

All data reported in this manuscript are found in the literature as cited in the text.

## Abbreviations

5FU: 5-Fluorouracil
CB: Carboplatin
ALT: Alanine aminotransferase
AST: Aspartate transaminase
C: Cisplatin
DCR: Disease control rate
DDD: Defined daily dose
DX: Dexamethasone
ECOG: Eastern Cooperative Oncology Group
E: Epirubicin
ET: Etoposide
FOLFIRI: 5-Fluorouracil and irinotecan
GC: Gemcitabine
GOJ: Gastro-oesophageal junction
GF: Gefitinib
HMG-CoA: Hydroxy-3-methylglutaryl-CoA
OS: Overall survival
Md: Median
Me: Mean
Mx: Maximum
NA: Non-adenocarcinomatous
NS: Not stated
PRISMA: Preferred Reporting Items for Systematic Reviews and Meta-Analyses
PFS: Progression-free survival
Rad R: Radiological response
RR: Response rate
RCT: Randomised controlled trial
TACE: Transcatheter arterial chemoembolisation
TTP: Time to progression
THL: Thalidomide
X: Capecitabine
XELIRI regimen: Capecitabine plus irinotecan
