# Supplementary material for: Efficacy and safety profile of statins in patients with cancer: a systematic review of randomised controlled trials
Source: Eur J Clin Pharmacol. 2020 Jul 28;76(12):1639–51. doi: 10.1007/s00228-020-02967-0 (PMC7661422; doi:10.1007/s00228-020-02967-0)
Supplement: Supplementary file 1 — (DOCX 13 kb) [file 228_2020_2967_MOESM1_ESM.docx]

| **Supplementary Table 1: Search string used in Embase and MEDLINE databases** | |
| --- | --- |
| **Searches** | **Results** |
| **1** | exp Hydroxymethylglutaryl-CoA Reductase Inhibitors/ |
| **2** | statin.mp. |
| **3** | cancer.mp. |
| **4** | Neoplasms/ |
| **5** | Carcinoma/ |
| **6** | malignancy.mp. |
| **7** | Clinical Trials as Topic/ |
| **8** | Randomized Controlled Trial/ |
| **9** | Randomized Controlled Trials as Topic/ |
| **10** | 1 or 2 |
| **11** | 3 or 4 or 5 or 6 |
| **12** | 7 or 8 or 9 |
| **13** | 10 and 11 and 12 |
| **14** | remove duplicates from 13 |
