# Supplementary material for: Efficacy and safety profile of statins in patients with cancer: a systematic review of randomised controlled trials
Source: Eur J Clin Pharmacol. 2020 Jul 28;76(12):1639–51. doi: 10.1007/s00228-020-02967-0 (PMC7661422; doi:10.1007/s00228-020-02967-0)
Supplement: Supplementary file 2 — (DOCX 16 kb) [file 228_2020_2967_MOESM2_ESM.docx]

| Supplementary Table 2: Participant characteristics in selected studies | | | | |  |  |
| --- | --- | --- | --- | --- | --- | --- |
|  | **Patients in statin group** | **Patients in control group** | **Age in statin group, mean ± SD; median (range)** | **Age in control group, mean ± SD; median (range)** | **Men in Statin group (%)** | **Men in control group (%)** |
| Alexandre et al 2019^21^ | 16 | 16 | 66.6 (8.7) | 62.7 (12.3) | 75% | 81.25% |
| Jouve et al 2019^22^ | 162 | 161 | 68 (37-86) | 68 (39-85) | 96.30% | 88.20% |
| Lee et al 2017^23^ | 36 | 32 | 59 (44-80) | 67 (44-78) | 88.90% | 84.40% |
| Seckl et al 2017^24^ | 422 | 424 | 64 (41-86) | 63 (42-85) | 51.90% | 50.50% |
| El-Hamamsy et al 2016^25^ | 25 | 25 | 53.6 ± 10.6 | 55.2 ± 11.8 | 44% | 56% |
| Lim et al 2015^26^ | 134 | 135 | 57.2 ± 9.41 | 57.1 ± 9.75 | 58.20% | 67.40% |
| Kim et al 2014^27^ | 120 | 124 | 53.5 (20-78) | 54.5 (24-79) | 75.80% | 68.50% |
| Hong et al 2014^28^ | 58 | 56 | 60 (38-80) | 56 (25-74) | 62.10% | 58.90% |
| Han et al 2011^29^ | 52 | 54 | 58 (20-76) | 60 (32-84) | 48% | 54% |
| Konings et al 2010^30^ | 15 | 15 | 59 (36-73) | 57 (42-74) | 73.30% | 86.70% |
| Kawata et al 2001^31^ | 41 | 42 | NS^a^ | NS^b^ | 76% | 80% |
| *NS* Not stated, *SD* Standard Deviation | |  |  |  |  |  |
| ^a^ Age < 60 = 37%, ≥ 60 = 63% | |  |  |  |  |  |
| ^b^ Age < 60 = 45%, ≥ 60 = 55% | |  |  |  |  |  |
