# Supplementary material for: Efficacy and safety profile of statins in patients with cancer: a systematic review of randomised controlled trials
Source: Eur J Clin Pharmacol. 2020 Jul 28;76(12):1639–51. doi: 10.1007/s00228-020-02967-0 (PMC7661422; doi:10.1007/s00228-020-02967-0)
Supplement: Supplementary file 4 — (DOCX 970 kb) [file 228_2020_2967_MOESM4_ESM.docx]

**
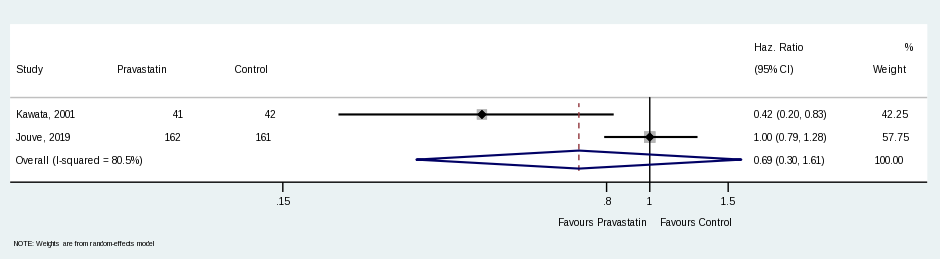
Supplementary Figure 1:** Forrest plot: All-cause mortality in patients with hepatocellular carcinoma allocated to pravastatin 40mg versus control
